# Risk of gastric cancer after bariatric surgery: a meta-analysis of retrospective studies

**DOI:** 10.3389/fsurg.2025.1573430

**Published:** 2025-06-13

**Authors:** Bing Li, Xiaoxuan Li, Jihong Peng, Pengfei Zhang

**Affiliations:** ^1^ Department of General Surgery, Yiling Hospital of Yichang City, Yichang, Hubei, China; ^2^ College of Basic Medical Sciences, China Three Gorges University, Yichang, Hubei, China

**Keywords:** bariatric and metabolic surgery MBS, gastric cancer, systermatic review, meta-analysis, review

## Abstract

**Objectives:**

As obesity rates rise and Bariatric & Metabolic surgery (MBS) becomes more common, many patients with obesity opt for these procedures. Despite this, there are still concerns regarding the risk of postoperative gastric cancer. This study reviews comparative studies on the risk of gastric gancer among MBS vs. non MBS patients, reported in the last 15 years.

**Methods and study design:**

We conducted literature searches on PubMed, Web of Science, and Cochrane Library using specifically formulated terms and limited the publication period to 2000 to 2024. The number of people in the literature who underwent MBS and those who developed gastric cancer after MBS were extracted and statistically analyzed using RevMan 5.3. A random-effects model was employed to determine the merged odds ratio (OR) values, with the Mantel-Haenszel estimation method. Publication bias was assessed using a funnel plot. Heterogeneity between studies was analyzed with the Cochran Q (Chi-square) test and I² statistics.

**Results:**

A total of nine studies reported the incidence of gastric cancer, with a total of 1,025,852 patients with obesity in the MBS group and 7,171,376 patients with obesity in the matched control group. After excluding the confounding factors commonly associated with gastric cancer in the included studies, we found that the incidence rate of gastric cancer was comparable for parents after MBS and patients with obesity (OR = 0.98, 95% CI 0.50–1.94, *P* = 0.96) in meta-analysis.

**Conclusions:**

It appears that there is no significant difference in the risk of gastric cancer between patients with obesity who have undergone MBS and those who have not, further investigation is needed to define the long term risk. Consequently, concerns can be reduced in patients with obesity who are in urgent need of MBS but are worried about developing gastric cancer. It provides evidence-based medicine evidence for clinical treatment.

## Introduction

Obesity is a chronic metabolic disease, reported by the World Health Organization to have more than doubled in adult obesity, and quadrupled in adolescents since 1990. Obesity is associated with an increased risk of cardiovascular diseases (1, 2), diabetes (2), respiratory issues (3), reproductive disorders, and other health problems (4). In addition, evidence indicates a causal relationship between obesity and several cancers (5, 6), including postmenopausal breast cancer (7), colorectal cancer (8), endometrial cancer (9), and gastrointestinal cancers (6). Therefore, overweight (bmi > 25) and obesity (bmi > 30) cannot be overlooked (10). treatment strategies for obesity include diet, exercising, medications, and MBS (11). MBS has been shown to be safe (12) and to achieve significantand long-term reduction of excess weight (13), and its associated comorbidities (14, 15). Importantly, weight loss surgery has been shown to improve cancer prognosis (15), especially breast, endometrial and ovarian cancers (16, 17).With the increasing prevalence of obesity and the widespread adoption of MBS, an increasing number of patients with obesity requiring weight loss surgery have undergone bariatric procedures.

However, there are still concerns about the risk of gastric cancer after bariatric surgery (18–21). The impact of MBS on gastric cancer incidence has been infrequently studied, with inconsistent results across different studies (16). Lazzati et al. (22) demonstrated that bariatric surgery is significantly associated with a reduced incidence of gastric cancer and an improvement in in-hospital mortality rates. Miller et al. (23) indicated that bariatric surgery lowers the overall risk of cancer occurrence and provides survival benefits, particularly when compared with similar patients who did not undergo surgery. Adams et al. (24) found that gastric bypass surgery may lead to a decreased incidence of cancer and cancer-related mortality, with more pronounced effects observed in women. Most studies support the positive role of bariatric surgery in reducing the risk of gastric cancer, suggesting it can be safely used in severely obese patients without significantly increasing the risk of gastric cancer. However, Esparham et al.'s research (25) presents a contrasting perspective on the risk of gastric cancer, emphasizing the need for further prospective studies to confirm these findings.

Therefore, to address this issue, we conducted a Meta-analysis.To our knowledge, this is the first meta-analysis on the risk of gastric cancer following MBS compared with patients who were not operated for obesity.

## Patients and methods

### Systematic review

For our literature search, we selected the following databases: PubMed, Web of Science, and the Cochrane Library, restricting the publication period to the years 2000 through 2024. Utilizing the search terms “MBS and gastric cancer”, “Roux-en-Y gastric bypass(RYGB) and gastric cancer”, “sleeve gastrectomy surgery(SG) and gastric cancer”, “Single anastomosis sleeve-ileal (SASI) and gastric Cancer”, “One anastomosis Gastric Bypass (OAGB) and gastric Cancer” or “Single anastomosis duodeno-ileal-sleeve (SADI-S) and gastric Cancer”, “Laparoscopic Adjustable Gastric Banding (LAGB) and gastric Cancer”, an initial screening yielded 190 articles from the major databases. Based on the article titles and abstracts, we excluded reviews, case reports, and conference proceedings. Ultimately, nine articles met our inclusion criteria; however, one was excluded due to incomplete gastric cancer data, resulting in a final dataset comprising eight articles for analysis. This study was not registered with PROSPERO. The research strictly adhered to relevant methodological guidelines (PRISMA for systematic reviews) to ensure transparency and rigor.

### Literature screening process

**Figure 1:**
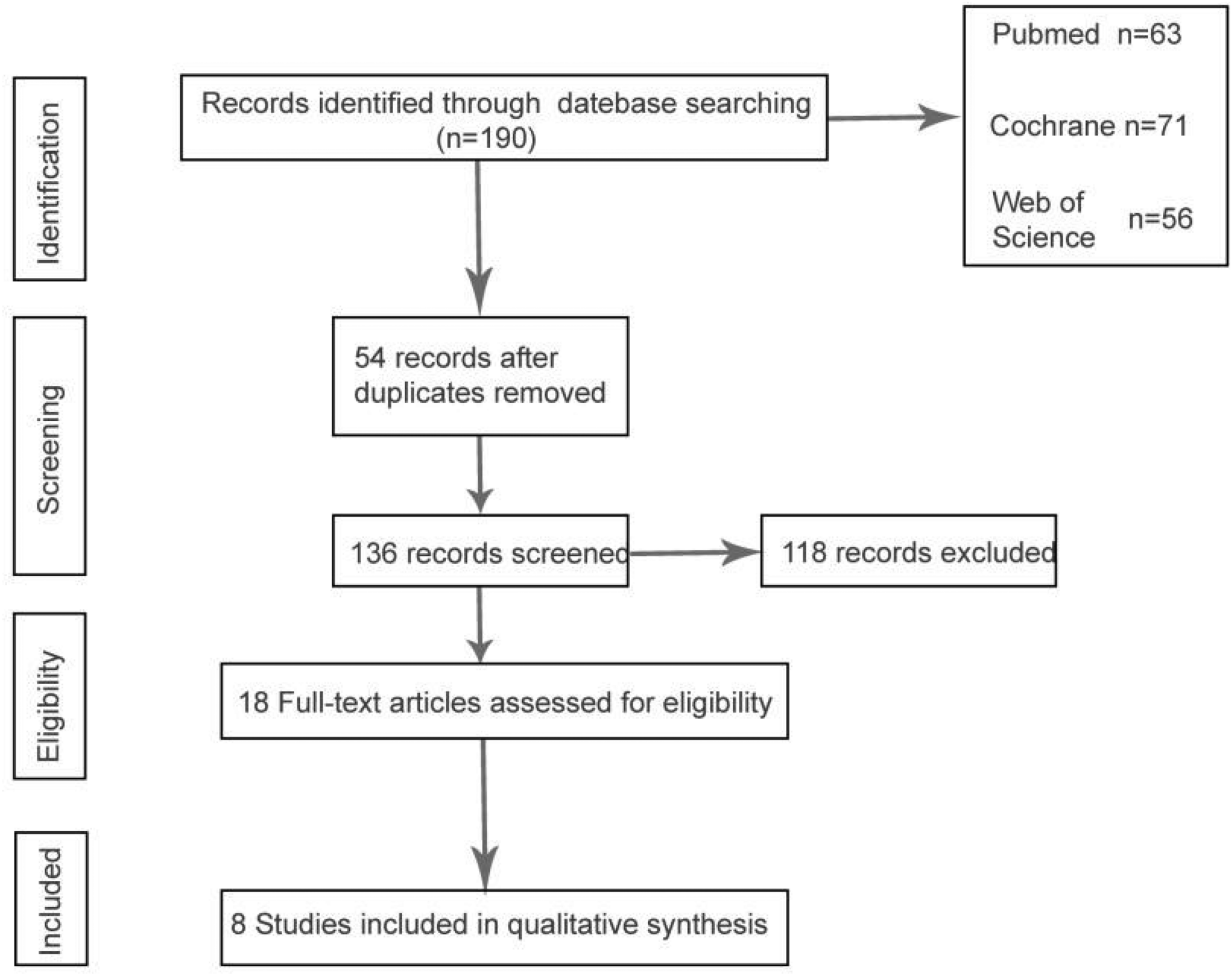
PRISMA flow chart.

According to the search strategy we established, the final search results are as follows. The full text of 18 articles was read carefully to exclude non-cohort studies and non-gastric cancer case groups, and a total of 8 cohort studies that met the criteria were included, one of which had missing data due to postoperative gastric cancer (26), and a total of 7 studies were analyzed by meta-analysis (23–25, 27–30), Figure 1 describes the process of article selection.

### Selection criteria

We reviewed the titles and abstracts of all searched articles to confirm that the patients underwent MBS. All citations that met the criteria were evaluated, and those repeated were removed. We further evaluated the article's relevance by reading the full text carefully, evaluating references in the article, and reviewing relevant reviews to locate additional candidate studies. We classified and managed all references by Endnote X9 (Research Software, Philadelphia, United States).

### Inclusion and exclusion criteria

The inclusion criteria were as follows: (1) adults ≥18 years old (2): randomized controlled trial or comparative cohort study of patients with obesity or morbid obesity that underwent MBS vs. controls. (3) the incidence of gastric cancer was investigated for at least 4 years after receiving the intervention. The exclusion criteria were as follows: (1) Literature type: case reports, conferences, responses, or animal experiments. (2) data on the incidence of gastric cancer after undergoing MBS was missing or not investigated. (3) Literature quality: literature scores were too low or did not meet the data standards. (4) duplicates.

### Data extraction and quality assessment

For accuracy, the two authors independently extracted data from eligible articles according to the inclusion and exclusion criteria and then discussed the results together. Specific data extracted included: investigator, time of publication and research, type of study, country of study, sample size, patient age, sex ratio, BMI criteria, and specific type of surgery. We used Review Manager (Version 5.3) to evaluate the risk of bias in these studies.

### Statistical analysis

The total number of people in the MBS and non-surgery groups and the number of people who developed gastric cancer in both groups after follow-up were first extracted in Excel tables. Then we performed the meta-analysis on the extracted data using RevMan 5.3. During the data analysis, since the outcome of interest (cancer occurrence) is a binary variable, we chose the Mantel-Haenszel method for analysis. A 95% confidence interval (CI) or a *p*-value of less than 0.05 was considered indicative of statistical significance for the included studies. Heterogeneity test: using *I*^2^ and *P*-value were tested, and there were two kinds of results: when *I*^2^ < 50% and *P* > 0.1, it indicated that there was homogeneity among the works of literature, and the fixed effect model was used; when *I*^2^ ≥ 50% and *P* ≤ 0.1, it indicated that there was heterogeneity among the literature, and the random effect model was used. We chose the random effects model because the data analysis showed *I*^2^ = 96% and *p*-value ≤ 0.1. We used RevMan 5.3 to create forest and funnel plots to statistically analyze the extracted data.

## Results

### Basic patient characteristics

The nine references we included are mainly retrospective studies published in recent years, with a wide range of study periods for the included patients and primarily focused on research from France and the United States. Among the studies on patients undergoing bariatric and metabolic surgery, three studies—Bariatric2023, Esparham2023 and Lazzati2022—had relatively large sample sizes. Specifically, the data for Bariatric2023 were derived from a French database, and the data for Esparham2023 were sourced from the National Inpatient Sample (NIS) database, the largest inpatient database in the United States. In terms of the included data across the studies, the average patient age mainly falls within the range of 40–60 years, and the gender ratio shows a predominance of male patients. Regarding the correlation between specific types of bariatric and metabolic surgery and the incidence of gastric cancer, only Khalid2022 and Tsui2020 provided detailed data, while the other studies did not clearly distinguish between different types of bariatric surgery. Table 1 summarizes the basic characteristics of the included studies and the participating patients.

**Table 1:** Characteristics of included studies.

Study	Study design	Study country	Included period	Number of cases treated	*N*	Gastric cancer events(incidence rate for gastric cancer)	*N*	Mean duration of follow-up (years)	*N*	Mean age (S vs. N)	Gender[male (%)/female (%)]	Definition of obesity (Kg/m²)	Operation
				S		S		S					
Adams et al. 2009 (24)	Retrospective	US	1984–2002	13,305	19,051	3 (0.023%)	4 (0.021%)	12.3	11.8	38.9 v 39.1	5.667 (85,15)	BMI ≥ 35	Roux-en-Y gastric bypass
Aminian et al. 2022 (26)	Retrospective	US	2004–2017	5,053	25,265	NA	NA	6.1	6.1	46.0 v 46.0	3.348 (77,23)	BMI ≥ 35	Roux-en-Y gastric bypass. sleeve gastrectomy
Bariatric 2023 (27)	Retrospective	France	NA	303,709	605,140	83 (0.027%)	254 (0.042%)	6	6	NA	NA	BMI ≥ 30	bariatric metabolic surgery
Esparham et al. 2023 (25)	Retrospective	US	2016–2020	328,369	489,154	259 (0.079%)	2,441 (0.50%)	NA	NA	55.35 v 56.71	1.632 (62,38)	BMI ≥ 30	bariatric metabolic surgery
Khalid et al. 2022 (28)	Retrospective	US	2010–2018	19,272	9,632	24 (0.12%)	7 (0.073%)	4	4	NA	4.596 (82,18)	BMI ≥ 30	vertical sleeve gastrectomy. Roux-en-Y gastric bypass
Lazzati et al. 2022 (29)	Retrospective	France	2010–2019	288,604	851,743	952 (0.33%)	3,918 (0.46%)	5.7	6.5	39.8 v 51.8	1.632 (62,38)	BMI ≥ 40	sleeve gastrectomy. gastric bypass
Miller et al. 2024 (23)	Retrospective	France	2001–2019	1,593	2,156	4 (0.25%)	1 (0.046%)	6.1	6	58.9 v 57.7	3.762 (79,21)	NA	Roux-en-Y gastric bypass. sleeve gastrectomy
Tsui et al. 2020 (30)	Retrospective	US	2006–2012	71,000	694,500	16 (0.023%)	120 (0.017%)	NA	NA	NA	NA	BMI ≥ 30	Roux-en-Y gastric bypass. sleeve gastrectomy. adjustable gastric banding

S, bariatric surgery; N, non bariatric surgery; v, versus; NA, not available.

### Data analysis of incidence rate for gastric cancer

**Figure 2:**
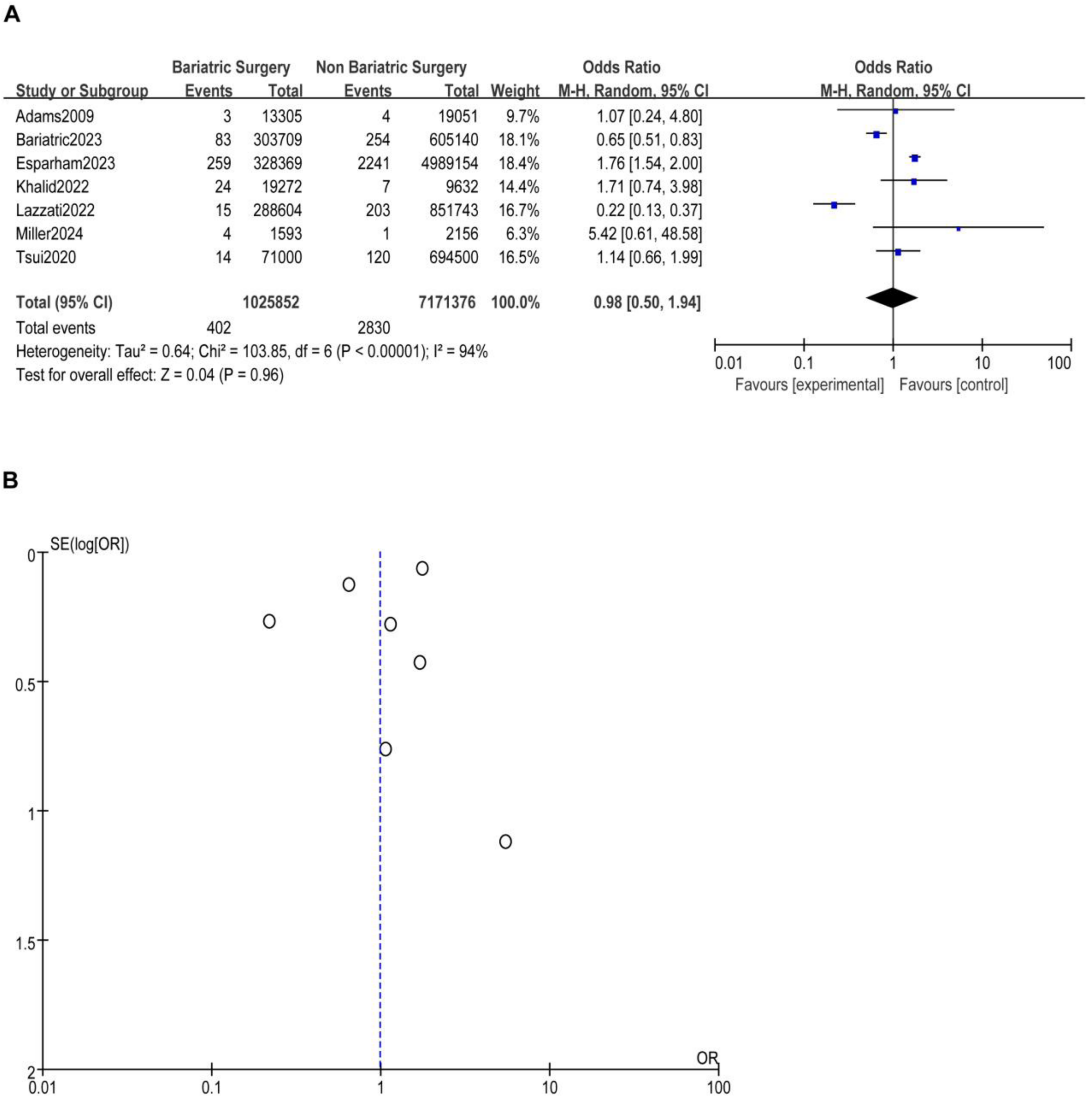
**(A)** The effect of bariatric vs. non-bariatric surgery in the development of gastric cancer. **(B)** Funnel plot for detecting and displaying system heterogeneity.

**Figure 3:**
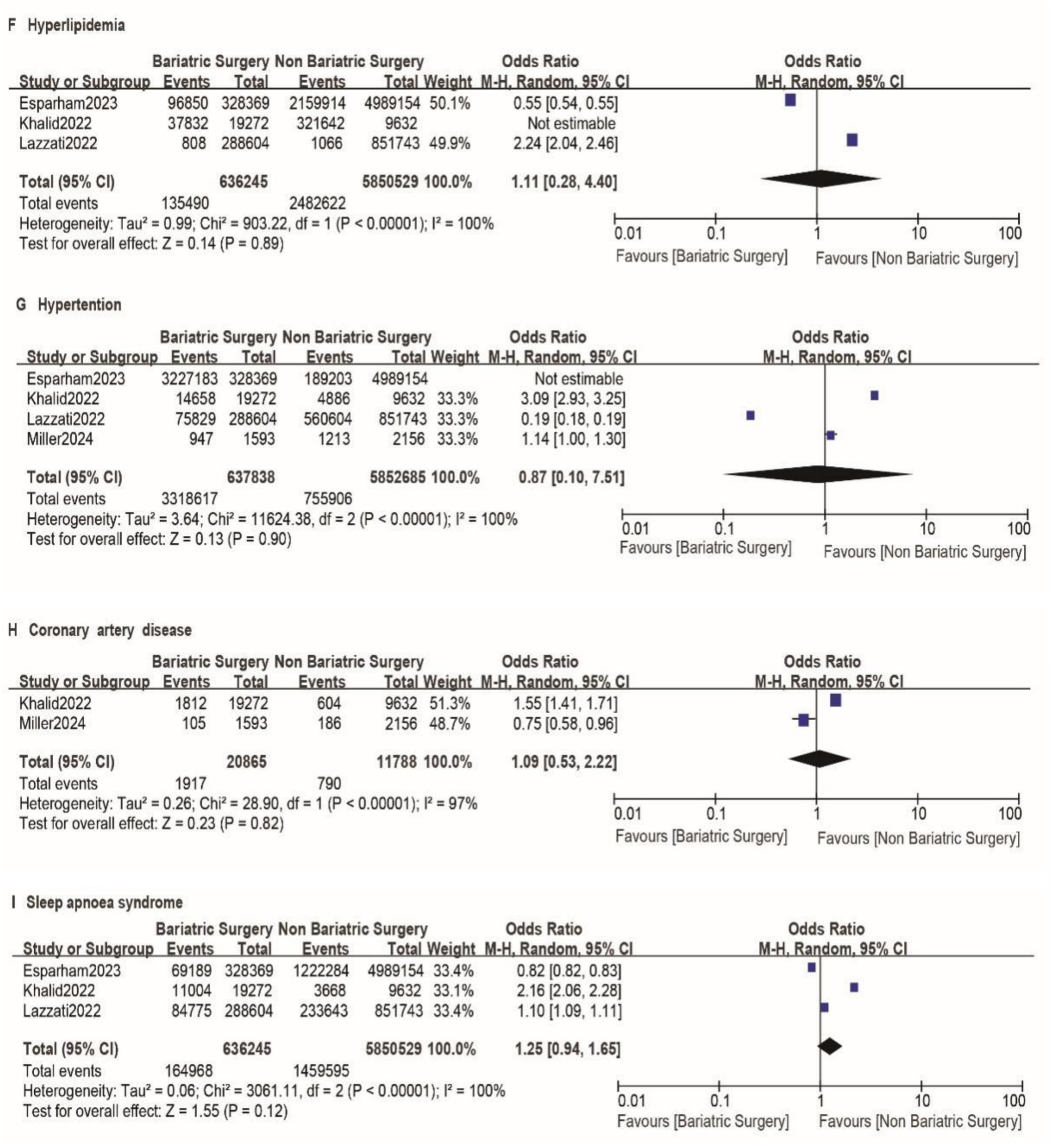
A-I impact of excluding patient risk factors on MBS. A-I represent the following risk factors, smoking, diabetes mellitus, chronic obstructive pulmonary disease, heart failure, myocardial infarction, hyperlipidemia, hypertension, coronary artery disease, sleep apnoea syndrome, respectively.

**Figure 4:**
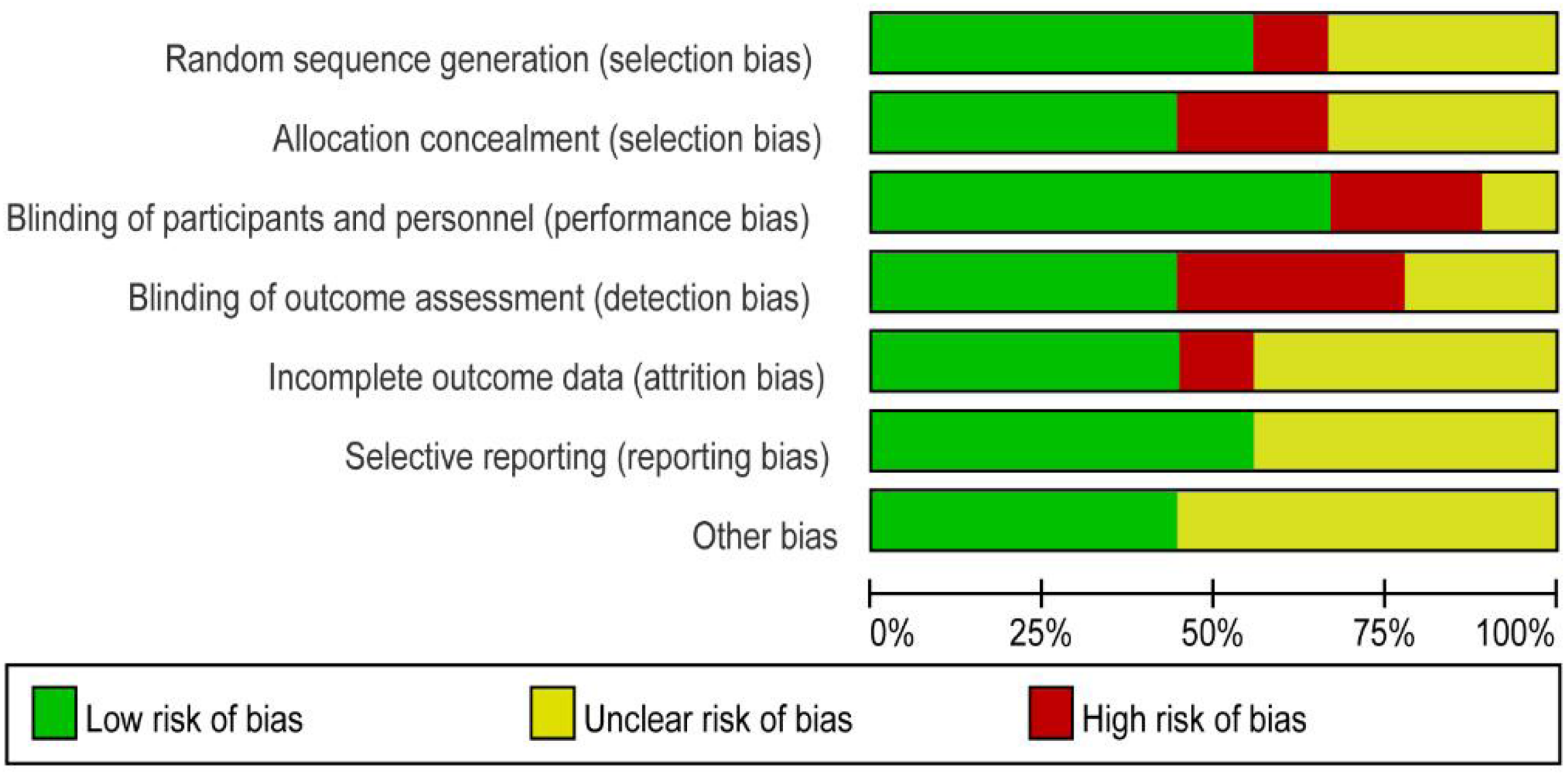
Risk of bias graph: the judgments about each risk of bias item are presented as percentages across all included studies.

**Figure 5:**
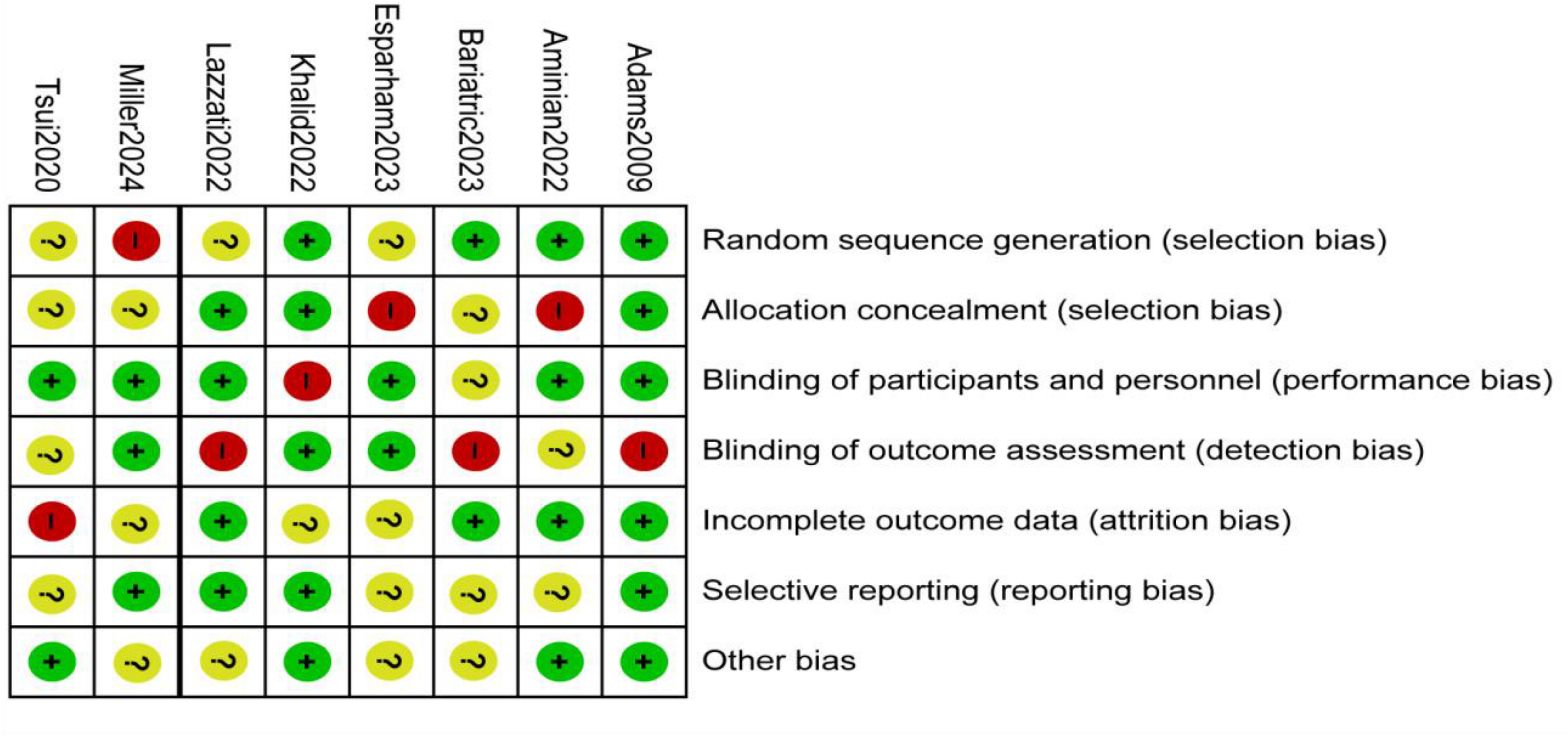
Risk of bias summary: the judgments about each risk of bias item for each included study.

A total of nine studies reported the incidence of gastric cancer, with a total of 1,025,852 patients with obesity in the MBS group and 7,171,376 patients with obesity in the matched control group. Statistically significant heterogeneity between studies is found by the Cochran Q test and I^2^ test (Chi 2 = 120.38, *P* < 0.00001, I^2^ = 94%), so a random-effects model is used. Meta-analysis shows that the incidence rate of gastric cancer after MBS is comparable to that of the non-operated patients with obesity (OR = 0.98, 95% CI 0.50- 1.94, *P* = 0.96), as shown in Figure 2A. The observed heterogeneity may be overestimated due to the small number of included studies. The results of the funnel plot in Figure 2B show that the distribution of points is close to the top suggesting a large sample size of included studies, and the included studies are more symmetrically distributed in the funnel plot. In addition, as shown in Figure 3, we excluded the influence of patients' relevant risk factors(smoking, diabetes mellitus, chronic obstructive pulmonary disease, heart failure, myocardial infarction, hyperlipidemia, hypertension, coronary artery disease, sleep apnoea syndrome,) on the association between MBS and gastric cancer. We used Revman to assess the risk of bias in the 9 included studies. In some of the included studies (24, 27, 29), the lack of blinding in outcome assessment led to unclear risk, which may introduce detection bias. Due to incomplete outcome data, some studies were rated as having a high risk (30) or unclear risk (22, 23, 25, 28). The individual and overall quality of studies in terms of bias is summarized in Figures 4, 5, respectively.

**Figure:**
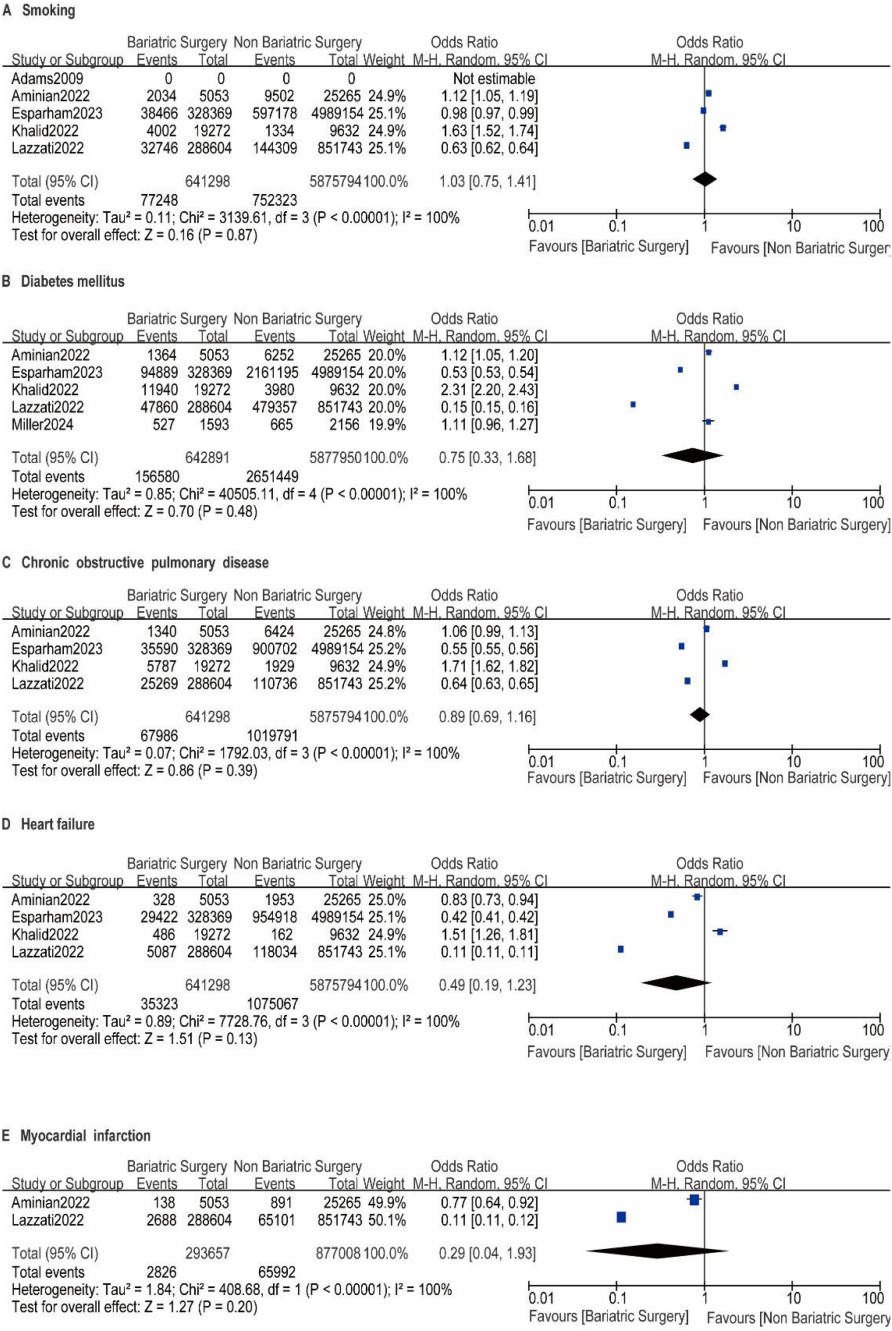


## Discussion

The International Agency for Research on Cancer (IARC) report indicates that when the BMI of patients with obesity exceeds 40, the relative risk of cardia cancer increases by 1.8%. The research results of Sung et al. show that obesity increases the risk of cardia cancer, with about 9% of cardia cancer in men and 11% in women attributable to obesity (31). Evidence shows a certain correlation between obesity and gastric cancer. With the rising prevalence of obesity and the increased adoption of MBS, more obese patients are opting for these procedures to lose weight. For patients with morbid obesity, MBS has been proven to be an important means of significantly reducing long-term weight, improving quality of life, and reducing the overall incidence and mortality of cancer in obese patients (5). However, currently, there are relatively few studies on the occurrence of gastric cancer after bariatric and metabolic surgery. Thus, the effect of MBS on the risk of postoperative gastric cancer has been a controversial topic (16).

To our knowledge, this is the first meta-analysis on the risk of gastric cancer following MBS. Our findings indicate that MBS does not increase the risk of postoperative gastric cancer in patients. MBS does not increase the risk of gastric cancer. The following reasons support this conclusion: First, sleeve gastrectomy removes a significant portion of the stomach, while gastric bypass surgery reduces the exposure of part of the stomach to food. Both procedures result in a marked reduction in gastric acid secretion. Excessive gastric acid is associated with chronic gastritis and gastroesophageal reflux disease (GERD), which can lead to long-term inflammation, particularly Helicobacter pylori-related gastric cancer. The decrease in gastric acid post-surgery may mitigate the risk of inflammation and mucosal damage. Second, obesity is an established independent risk factor for various cancers, including gastric cancer. Bariatric surgery effectively reduces body weight and improves metabolic parameters, thereby diminishing the obesity-associated pro-carcinogenic environment. Additionally, the reduced stomach capacity after surgery may decrease food retention time, further reducing the likelihood of mucosal injury. Finally, patients undergoing MBS are subject to long-term follow-up, during which diagnostic tools such as gastroscopy can facilitate early detection and intervention of precancerous lesions.

Regarding the types of bariatric metabolic surgery, Roux-en-Y gastric bypass is the primary procedure, followed by sleeve gastrectomy (SG), which is consistent with the study by Chetan Parmar et al. (32). As for the location of gastric cancer, only the studies by Esparham2023 (25) and Lazzati2023 (22) clearly mentioned that gastric cancer mainly occurs in the body, pylorus, and antrum of the stomach. Other studies did not specify the exact location of gastric cancer, likely because their focus was not on the anatomical distribution of gastric cancer. However, the location of gastric cancer after surgery also needs to be paid more attention by researchers. We have excluded confounding factors related to gastric cancer risk in the included literature, thereby reducing the impact of these factors on the outcomes. This study provides a higher level of evidence-based medical evidence for patients who urgently need MBS but are concerned about the risk of developing gastric cancer postoperatively, aiding clinicians in making further decisions.

The results of some comprehensive analytical studies are described as follows. Lim et al. (16) pointed out that, as sleeve gastrectomy has only been progressively adopted in the last few years, the impact of MBS on the risk of gastric cancer remains uncertain. Longer follow-up data are needed to clarify the correlation between MBS and the risk of gastric cancer. Stella et al. (30) found that there are no statistically significant differences between the three different types of MBS for postoperative obesity-related cancers, this is in line with our findings. Mario Musella et al. (33) found that there is currently insufficient evidence to demonstrate a significant association between bariatric surgery and gastric cancer. However, it is necessary to conduct long-term follow-up of patients who have undergone bariatric surgery to monitor any new or altered upper gastrointestinal symptoms. Chetan Parmar et al. (32) found that the majority of patients had Roux-en-Y gastric bypass (RYGB) as their primary bariatric surgery, followed by gastric banding (GB) and sleeve gastrectomy (SG). Although it is not possible to determine the exact incidence rate, the study results suggest that the occurrence of gastric cancer after bariatric surgery is not high. Sotirios G. Doukas et al. (18) assessed the occurrence of gastric cancer after bariatric bypass surgery and explored the potential association between them.

However, there are still some limitations in our study. The studies included are retrospective cohort studies, with the majority of patients recruited from the United States and France, and most are middle-aged. There are limitations in the selection of study methods, regions, and age groups of patients due to varying dietary habits, economic conditions, and regional disparities, which affect the risk of gastric cancer and patients’ acceptance of MBS.

Therefore, In the future clinical studies, we advocate for more global scholars to conduct multicenter randomized controlled clinical studies to investigate the risk of cancer following MBS, which will benefit patient prognosis, particularly in light of the potential influence of different surgical procedures (e.g., sleeve gastrectomy) on cancer risk. During the design of clinical studies, we recommend that researchers fully assess the preoperative gastric conditions of patients undergoing bariatric and metabolic surgery whenever possible. In the follow-up process, it is important to clearly document the pathological conditions and locations of cancer in patients. This information will be helpful for other scholars to conduct in-depth research. Additionally, detailed recording of the different surgical procedures and the number of patients who develop cancer will also assist clinicians in selecting appropriate surgical methods and provide some guidance for clinical decision-making.

## Conclusion

As far as this study is concerned, there is no difference in the incidence risk of gastric cancer in patients with obesity between bariatric and non-bariatric surgery, so concerns can be reduced in patients with obesity who are in urgent need of MBS but are worried about developing gastric cancer. It provides evidence-based medicine evidence for clinical treatment.

## Data Availability

The original contributions presented in the study are included in the article/Supplementary Material, further inquiries can be directed to the corresponding author.
